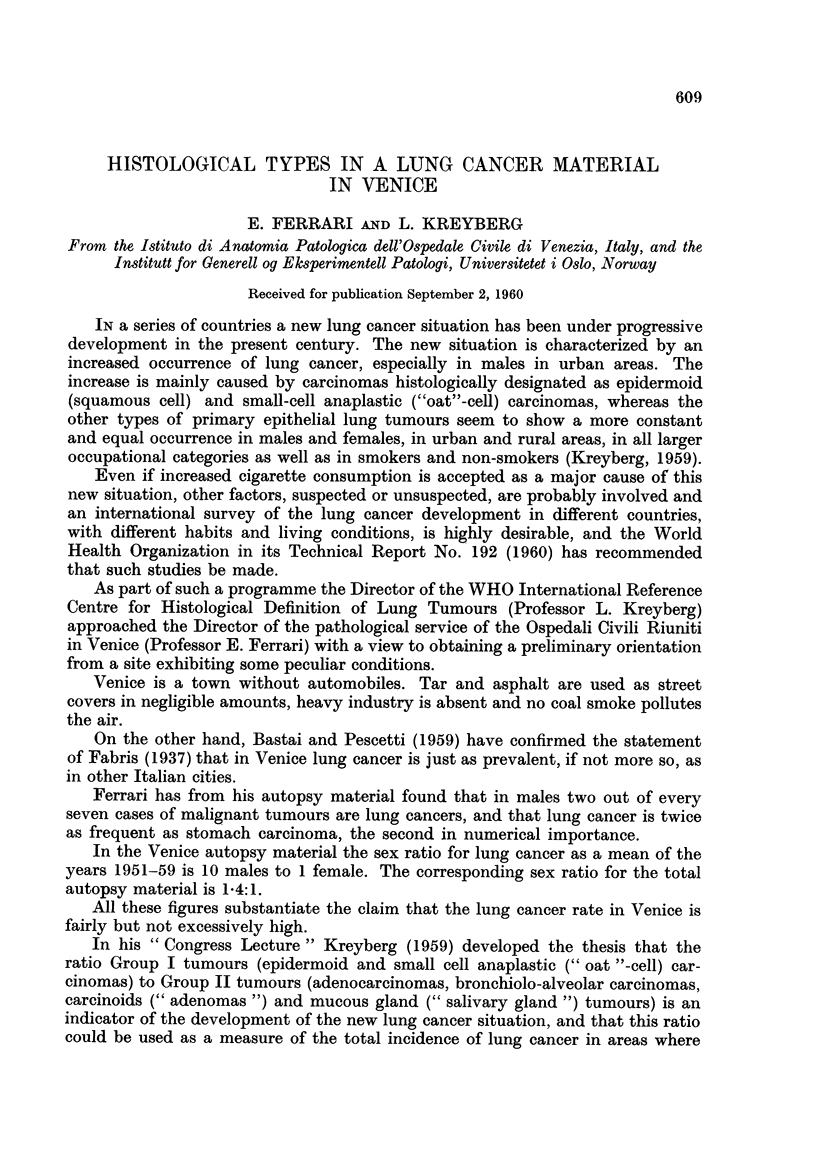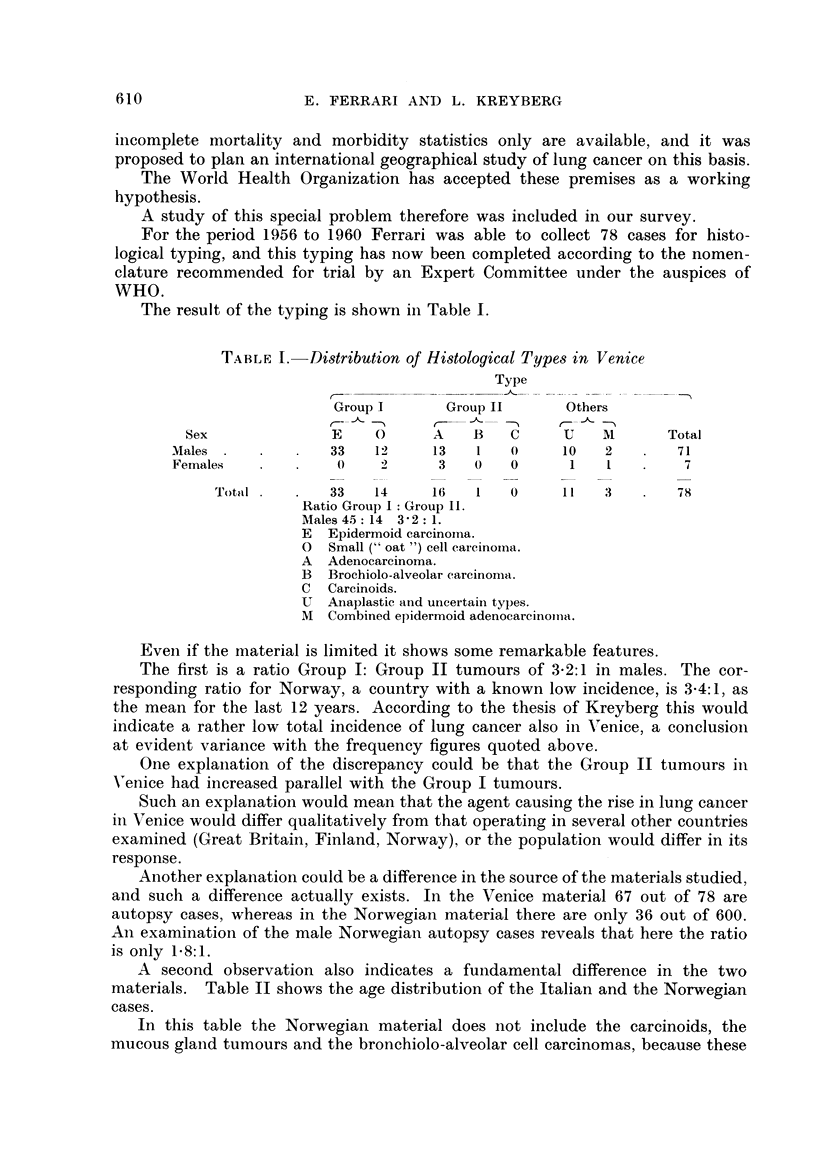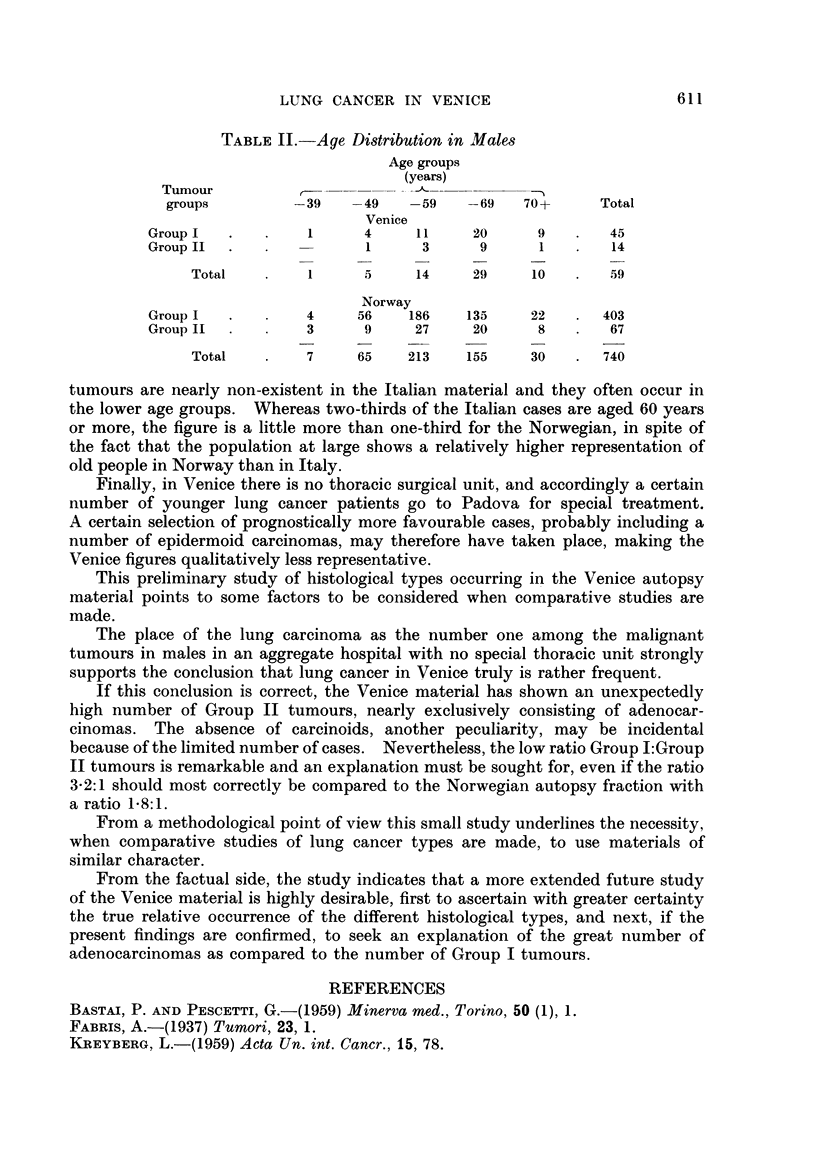# Histological Types in a Lung Cancer Material in Venice

**DOI:** 10.1038/bjc.1960.66

**Published:** 1960-12

**Authors:** E. Ferrari, L. Kreyberg


### HISTOLOGICAL TYPES IN A LUNG CANCER MATERIAL IN VENICE

E. FERRARI AND L. KREYBERG

*From the Istituto di Anatomia Patologica dell'Ospedale Civile di Venezia, Italy, and the Institutt for Generell og Eksperimentell Patologi, Universitetet i Oslo, Norway*

Received for publication September 2, 1960

IN a series of countries a new lung cancer situation has been under progressive development in the present century. The new situation is characterized by an increased occurrence of lung cancer, especially in males in urban areas. The increase is mainly caused by carcinomas histologically designated as epidermoid (squamous cell) and small-cell anaplastic ("oat"-cell) carcinomas, whereas the other types of primary epithelial lung tumours seem to show a more constant and equal occurrence in males and females, in urban and rural areas, in all larger occupational categories as well as in smokers and non-smokers (Kreyberg, 1959).

Even if increased cigarette consumption is accepted as a major cause of this new situation, other factors, suspected or unsuspected, are probably involved and an international survey of the lung cancer development in different countries, with different habits and living conditions, is highly desirable, and the World Health Organization in its Technical Report No. 192 (1960) has recommended that such studies be made.

As part of such a programme the Director of the WHO International Reference Centre for Histological Definition of Lung Tumours (Professor L. Kreyberg) approached the Director of the pathological service of the Ospedali Civili Riuniti in Venice (Professor E. Ferrari) with a view to obtaining a preliminary orientation from a site exhibiting some peculiar conditions.

Venice is a town without automobiles. Tar and asphalt are used as street covers in negligible amounts, heavy industry is absent and no coal smoke pollutes the air.

On the other hand, Bastai and Pescetti (1959) have confirmed the statement of Fabris (1937) that in Venice lung cancer is just as prevalent, if not more so, as in other Italian cities.

Ferrari has from his autopsy material found that in males two out of every seven cases of malignant tumours are lung cancers, and that lung cancer is twice as frequent as stomach carcinoma, the second in numerical importance.

In the Venice autopsy material the sex ratio for lung cancer as a mean of the years 1951–59 is 10 males to 1 female. The corresponding sex ratio for the total autopsy material is 1·4:1.

All these figures substantiate the claim that the lung cancer rate in Venice is fairly but not excessively high.

In his " Congress Lecture " Kreyberg (1959) developed the thesis that the ratio Group I tumours (epidermoid and small cell anaplastic (" oat "-cell) carcinomas) to Group II tumours (adenocarcinomas, bronchiolo-alveolar carcinomas, carcinoids (" adenomas ") and mucous gland (" salivary gland ") tumours) is an indicator of the development of the new lung cancer situation, and that this ratio could be used as a measure of the total incidence of lung cancer in areas where incomplete mortality and morbidity statistics only are available, and it was proposed to plan an international geographical study of lung cancer on this basis.

The World Health Organization has accepted these premises as a working hypothesis.

A study of this special problem therefore was included in our survey.

For the period 1956 to 1960 Ferrari was able to collect 78 cases for histological typing, and this typing has now been completed according to the nomenclature recommended for trial by an Expert Committee under the auspices of WHO.

The result of the typing is shown in Table I.

TABLE I.—*Distribution of Histological Types in Venice*

| | Type | | | | | | | |
|---|---|---|---|---|---|---|---|---|
| | Group I | | Group II | | | Others | | |
| Sex | E | O | A | B | C | U | M | Total |
| Males | 33 | 12 | 13 | 1 | 0 | 10 | 2 | 71 |
| Females | 0 | 2 | 3 | 0 | 0 | 1 | 1 | 7 |
| Total | 33 | 14 | 16 | 1 | 0 | 11 | 3 | 78 |

Ratio Group I : Group II.
Males 45 : 14 3·2 : 1.
E Epidermoid carcinoma.
O Small (" oat ") cell carcinoma.
A Adenocarcinoma.
B Brochiolo-alveolar carcinoma.
C Carcinoids.
U Anaplastic and uncertain types.
M Combined epidermoid adenocarcinoma.

Even if the material is limited it shows some remarkable features.

The first is a ratio Group I: Group II tumours of 3·2:1 in males. The corresponding ratio for Norway, a country with a known low incidence, is 3·4:1, as the mean for the last 12 years. According to the thesis of Kreyberg this would indicate a rather low total incidence of lung cancer also in Venice, a conclusion at evident variance with the frequency figures quoted above.

One explanation of the discrepancy could be that the Group II tumours in Venice had increased parallel with the Group I tumours.

Such an explanation would mean that the agent causing the rise in lung cancer in Venice would differ qualitatively from that operating in several other countries examined (Great Britain, Finland, Norway), or the population would differ in its response.

Another explanation could be a difference in the source of the materials studied, and such a difference actually exists. In the Venice material 67 out of 78 are autopsy cases, whereas in the Norwegian material there are only 36 out of 600. An examination of the male Norwegian autopsy cases reveals that here the ratio is only 1·8:1.

A second observation also indicates a fundamental difference in the two materials. Table II shows the age distribution of the Italian and the Norwegian cases.

In this table the Norwegian material does not include the carcinoids, the mucous gland tumours and the bronchiolo-alveolar cell carcinomas, because these

TABLE II.—*Age Distribution in Males*

| Tumour groups | Age groups (years) −39 | −49 | −59 | −69 | 70+ | Total |
|---|---|---|---|---|---|---|
| | | Venice | | | | |
| Group I | 1 | 4 | 11 | 20 | 9 | 45 |
| Group II | — | 1 | 3 | 9 | 1 | 14 |
| Total | 1 | 5 | 14 | 29 | 10 | 59 |
| | | Norway | | | | |
| Group I | 4 | 56 | 186 | 135 | 22 | 403 |
| Group II | 3 | 9 | 27 | 20 | 8 | 67 |
| Total | 7 | 65 | 213 | 155 | 30 | 740 |

tumours are nearly non-existent in the Italian material and they often occur in the lower age groups. Whereas two-thirds of the Italian cases are aged 60 years or more, the figure is a little more than one-third for the Norwegian, in spite of the fact that the population at large shows a relatively higher representation of old people in Norway than in Italy.

Finally, in Venice there is no thoracic surgical unit, and accordingly a certain number of younger lung cancer patients go to Padova for special treatment. A certain selection of prognostically more favourable cases, probably including a number of epidermoid carcinomas, may therefore have taken place, making the Venice figures qualitatively less representative.

This preliminary study of histological types occurring in the Venice autopsy material points to some factors to be considered when comparative studies are made.

The place of the lung carcinoma as the number one among the malignant tumours in males in an aggregate hospital with no special thoracic unit strongly supports the conclusion that lung cancer in Venice truly is rather frequent.

If this conclusion is correct, the Venice material has shown an unexpectedly high number of Group II tumours, nearly exclusively consisting of adenocarcinomas. The absence of carcinoids, another peculiarity, may be incidental because of the limited number of cases. Nevertheless, the low ratio Group I:Group II tumours is remarkable and an explanation must be sought for, even if the ratio 3·2:1 should most correctly be compared to the Norwegian autopsy fraction with a ratio 1·8:1.

From a methodological point of view this small study underlines the necessity, when comparative studies of lung cancer types are made, to use materials of similar character.

From the factual side, the study indicates that a more extended future study of the Venice material is highly desirable, first to ascertain with greater certainty the true relative occurrence of the different histological types, and next, if the present findings are confirmed, to seek an explanation of the great number of adenocarcinomas as compared to the number of Group I tumours.

### REFERENCES

BASTAI, P. AND PESCETTI, G.—(1959) *Minerva med., Torino*, **50** (1), 1.
FABRIS, A.—(1937) *Tumori*, **23**, 1.
KREYBERG, L.—(1959) *Acta Un. int. Cancr.*, **15**, 78.